# Accuracy of one-dimensional templating on linear EOS radiography allows template-directed instrumentation in total knee arthroplasty

**DOI:** 10.1186/s13018-021-02812-9

**Published:** 2021-11-10

**Authors:** Michael Andreas Finsterwald, Salar Sobhi, Senthuren Isaac, Penelope Scott, Riaz J. K. Khan, Daniel P. Fick

**Affiliations:** 1 The Joint Studio, Hollywood Medical Centre, 85 Monash Avenue, Nedlands, WA 6009 Australia; 2 Hollywood Private Hospital, Monash Avenue, Nedlands, WA 6009 Australia; 3 Faculty of Science and Engineering, Curtin University, Kent Street, Bentley, WA 6102 Australia; 4 School of Medicine, University of Notre Dame, 9 Mouat Street, Fremantle, WA 6959 Australia

**Keywords:** Template-directed instrumentation, Total knee arthroplasty, Total knee replacement, One-dimensional templating, Tray reduction, Cost reduction, Cost analysis

## Abstract

**Background:**

Templating for total knee arthroplasty (TKA) is routinely performed on two-dimensional standard X-ray images and allows template-directed instrumentation. To date, there is no report on one-dimensional (1D) anteroposterior (AP) templating not requiring specific templating software. We aim to describe a novel technique and explore its reliability, accuracy and potential cost-savings.

**Methods:**

We investigated a consecutive series of TKAs at one institution between January and July 2019. Patients with preoperative low-dose linear AP EOS radiography images were included. Implant component sizes were retrospectively templated on the AP view with the hospitals imaging viewing software by two observers who were blinded to the definitive implant size. Planning accuracy as well as inter- and intra-observer reliability was calculated. Cost-savings were estimated based on the reduction of trays indicated by the 1D templating size estimations.

**Results:**

A total of 141 consecutive TKAs in 113 patients were included. Accuracy of 1D templating was as follows: exact match in 53% femoral and 63% tibial components, within one size in 96% femoral and 98% tibial components. Overall 58% of TKA components were planned correctly and 97% within one size. Inter- and intra-rater reliability was good (*κ* = 0.66) and very good (*κ* = 0.82), respectively. This templating process can reduce instrumentation from six to three trays per case and therefore halve sterilisation costs.

**Conclusions:**

The new 1D templating method using EOS AP imaging predicts component sizes in TKA within one size 97% of the time and can halve the number of instrumentation trays and sterilisation costs.

## Introduction

Preoperative planning is of great importance in TKA surgery, regardless of which technique is undertaken [1, 2]. It allows operating staff to prepare and ready component sizes, gives the surgeon an idea of what to expect intraoperatively, and can influence on the long-term success rate of TKA [3]. In the past decades, templating for TKA and total hip arthroplasty (THA) was performed on printed radiographic films with a set magnification factor and acetate overlays [4, 5]. With the introduction of digital imaging came the development of digital planning software, which allowed for implant sizes to be templated digitally with comparable accuracy to using acetate overlays [4, 6]. For TKA, the current standard of practice employs a two-dimensional (2D) process, whereby AP and lateral X-ray images of the knee are undertaken with a reference ball to achieve higher accuracy [6]. Good inter- and intra-observer reliability has been reported for 2D templating among different levels of training [7]. In the last decade, patient-specific instrumentation (PSI) was developed. This mandates acquisition of three-dimensional (3D) imaging, which is achieved by computed tomography scan (CT) or magnetic resonance imaging (MRI) to allow for production of patient-specific cutting blocks. It has been shown that 3D exceeds 2D methods of templating in terms of accuracy [8] and reduces number of trays used [9]. The process, however, has the drawbacks of producing cutting blocks, which is time-consuming and costly compared to conventional instrumentation and has not yielded better patient outcomes, limb alignment or cost-effectiveness [10]. Linear radiography EOS 2D-3D imaging system (EOS Imaging, Paris, France) on the other hand has the advantage in that only low-dose biplanar X-ray images of the patient in a weight-bearing upright position [11] need to be acquired. A 1:1 scale removes magnification or distortion errors, including in the obese patient [12], and therefore makes a calibration ball redundant [13]. Furthermore, synchronised acquisition of AP and lateral images allows for 3D reconstructions of lower limb torsion measurements with the precision of standard CT scans [14]. Additionally, low-dose EOS imaging protocols reduce radiation compared to conventional radiographs twofold in lower limb measurements [15]. The system has been shown to be accurate and reliable in templating THA in 2D or with 3D reconstructions [16]; however, reports on TKA templating are lacking. Furthermore, while component size estimations based on demographic variables [17–19] and shoe size [20, 21] have been presented, our paper is the first to report on 1D digital AP templating in TKA.

Template-directed instrumentation (TDI) is a term describing a process introduced by Hsu et al. [22] in 2012 with the goal to reduce the number of conventional instrumentation trays needed for TKA. While Hsu et al. [22] showed that TDI could reduce average number of trays by 60% and save on sterilisation expenses, further studies [23] have examined the impact on the economics and operating room (OR) time in more detail. It was reported that TDI reduces costs and improves OR efficiency by a significant reduction of OR turnover time and in-room time [23]. The same authors stated that with a planning accuracy threshold of 50% in their cohort and 57.3% in the literature, TDI can be favoured over non-TDI regarding costs [23].

The primary objective of our study was to determine the accuracy and reliability of 1D templating using solely digital AP views of the EOS radiographs in the setting of TKA and whether this method would allow TDI. The secondary objective was to analyse potential cost-savings of TDI based on this 1D templating in our institution.

## Methods

The study protocol was deemed ethically sound by our hospital ethical committee and all patients gave informed consent to participate. Patients were included if they received primary TKA by one of the two arthroplasty surgeons (<blinded>) between January and July 2019 and had a preoperative standing whole-leg biplanar EOS scan. Biplanar AP and lateral EOS low-dose X-rays were taken at one institution (SKG Radiology, Subiaco, Australia) at least one week prior to surgery. Patient demographics of age, gender and laterality of the procedure, and the definitive implant sizes were derived from the medical records. The only exclusion criterion was the absence of a preoperative EOS scan.

TKA were implanted in a standardised fashion through a medial parapatellar approach. All femoral components were uncemented, tibial components were cemented or uncemented according to senior surgeons’ (<blinded>) discretion. Cemented patella resurfacing was performed in 90% of the cases. The implant used was the mobile bearing Score® total knee prosthesis (Amplitude, Valence, France). Anterior referencing was used for femoral sizing and rotation. Intramedullary instrumentation was used for proximal tibial and distal femoral cuts. Standard number of trays used in conventional TKA in our hospital is 6 with and 5 without patella tray.

Retrospectively, digital EOS radiographs were accessed with the hospitals picture archiving and communication system (IntelePACS) and measured with its installed software (InteleViewer, Intelerad Medical Systems Incorporated, Montreal, Canada) by two raters. The first author (<blinded>), an orthopaedic fellow with > 5-year templating experience, and a blinded medical student (<blinded>), who was new to templating and instructed on how to perform the measurements. The two raters were blinded to each others sizing estimates, and the definitive size that had been surgically implanted. Repeated templating was undertaken by both researchers after a period of six weeks.

**Fig. 1:**
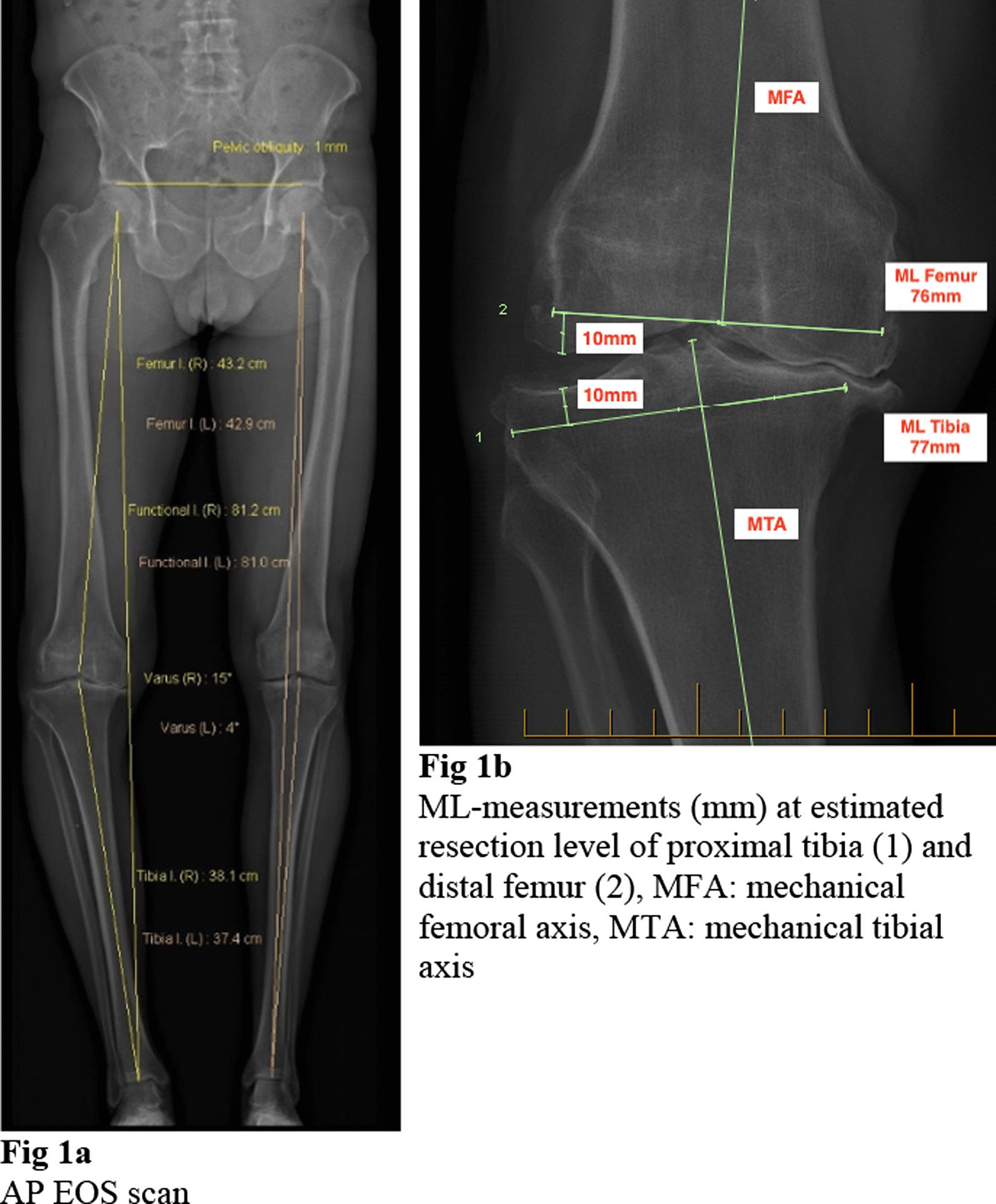
**a** AP EOS scan. **b** ML measurements (mm) at estimated resection level of proximal tibia (1) and distal femur (2), MFA: mechanical femoral axis, MTA: mechanical tibial axis

Measurements were performed on AP views only due to the rotational variance on the simultaneously acquired lateral views, which is why the method was named one-dimensional (1D) templating. It required an AP EOS scan (Fig. 1a) and consisted of mediolateral (ML) measurement in millimetres (mm) at the height of the planned proximal tibial and distal femoral cut. Distal femoral cut was measured perpendicular to the mechanical femoral axis (MFA) with a planned resection of 10 mm from the less worn condyle to account for the implant thickness. This roughly corresponded to the roof of the femoral notch. The ML measurement was guided by the sclerotic lines of the medial and lateral femoral condyle while ignoring osteophytes. The proximal tibial cut was set perpendicular to the anatomical and mechanical tibial axis and a 10 mm resection from the healthy tibial plateau planned while ignoring osteophytes (Fig. 1b). ML measurements were recorded and translated into a tibial and femoral component size according to the companies sizing chart with 3.3–3.4 mm increments between femoral and 3.5 mm increments between tibial component sizes (Table 1). Implanted sizes were recorded and compared to templated sizes. The number of trays that the 1D EOS templating process deemed as required, was also recorded.

**Table 1:** Mediolateral (ML) component sizes Score® total knee prosthesis (Amplitude, Valence, France)

Femur component size	1	2	3	4	5	6	7
ML measurement (mm)	60	63.3	66.7	70	73.3	76.7	80
Tibia component size	1	2	3	4	5	6	7
ML measurement (mm)	63.5	67	70.5	74	77.5	81	84.5

The 1D EOS templated sizes were compared to the implanted femoral and tibial component sizes, also the number of surgical trays used. Potential savings of TDI based on our retrospective templating audit were acquired from the hospital’s sterilisation department and included cleaning, sterilisation and labour costings.

### Statistics

Templated femoral and tibial component sizes were compared to implanted sizes with the Wilcoxon signed rank test. Percentages for exact match as well as plus or minus one size were calculated. Cohen's kappa coefficient (*κ*) was calculated for inter- and intra-rater agreement of measurements. Table 2 interprets (*κ*) as level of agreement [24]. Significance level was set at *p* < 0.05. Cost analysis was evaluated using the Mann–Whitney *U * test.

**Table 2:** Cohen's kappa coefficient (*κ*) and level of agreement [22]

Range of *κ*	< 0.20	0.21 to 0.40	0.41 to 0.60	0.61 to 0.80	0.81 to 1.00
Level of agreement	Poor	Fair	Moderate	Good	Very good

## Results

### Patients

A total of 141 TKAs in 113 patients (52 male, 61 female) were templated using the 1D EOS digital system. The cohort mean age was 68 years (range, 47–94, SD 9.5). Further demographics are described in Table 3. The surgical demographic was 28 bilateral, 71 left and 70 right TKA. No patients had to be excluded.

**Table 3:** Demographics

Demographics	Patients (*n* = 113)
Age (Years)	67.9 (range, 47–94, SD 9.5)
Gender	
Male	52 (46%)
Female	61 (54%)
Side	
Left (*n*)	43 (38%)
Right (*n*)	42 (37%)
Bilateral (*n*)	28 (25%)

### Templating accuracy

In total 564 femoral and 564 tibial component measurements were undertaken. Exact match for femoral and tibial component sizes was achieved in 53% (75/141) and 63% (89/141), respectively. Femoral and tibial components were templated within one size of the implanted component in 96% (136/141) and 98% (138/141), respectively. Overall 58% (164/282) of components were templated correctly and 97% (274/282) within one size. In five knees (3%), femoral component was templated two sizes too small and in three (2%) of these the same error occurred for tibial templating [20]. In 93% (26/28) of the bilateral cases, sizes matched the other side and in only two cases the tibial and femoral component each differed by one size.

### Templating reliability

Intra-rater agreement was very good for both the orthopaedic fellow and the medical student (Table 4). Mean inter-rater agreement was good for femoral and tibial components (Table 5).

**Table 4:** Weighted κ intra-observer coefficients

Observer	*κ*	95% CI*
Femur		
Fellow	0.84	0.78–0.91
Student	0.84	0.78–0.91
Mean	0.84	0.78–0.91
Tibia		
Fellow	0.83	0.76–0.9
Student	0.76	0.68–0.84
Mean	0.8	0.72–0.87

*95% confidence interval

**Table 5:** Weighted *κ* inter-observer coefficients

Observer	κ	95% CI*
Femur		
Mean	0.67	0.58–0.75
Tibia		
Mean	0.66	0.57–0.75

*95% confidence interval

### Cost analysis

Based on our templating accuracy, in 136 of the 141 TKA cases (97%), TDI would have allowed surgery to be conducted with two size and side-specific trays and an extra patella tray, which was used in 90%. In 5 cases (3%), TDI would have failed due to a femoral or tibial component size planning error of two sizes, which in turn necessitates opening two extra trays. Average number of trays with TDI would have been 3 (range, 2–5) versus 6 (range, 5–6) in non-TDI conventional TKA. Total sterilisation costs per tray at our hospital were $78 AUD ($53 USD). Total cost for 141 TDI cases would have been $31,902 AUD ($21,702 USD) for a total of 409 trays (90% with Patella: 127 × 3 = 381, 10% without Patella:14 × 2 = 28), averaging $226 AUD/case ($155 USD). Total sterilisation cost of 141 conventional cases was $64,896 AUD ($44,146 USD) for a total of 832 trays (90% with Patella: 127 × 6 = 762, 10% without Patella:14 × 5 = 70), averaging $460 AUD/case ($315 USD). Introduction of TDI would therefore allow for a significant reduction of trays per case by 50% (6 to 3) (*p* < 0.00001) and a reduction of sterilisation costs per case by 50% (*p* < 0.00001).

## Discussion

This study highlights a novel and simple technique of 1D templating of TKA component size that uses linear low-dose weight-bearing whole body AP EOS radiographs. Our data reveals that 1D templating has 97% accuracy to predict component sizes within one. This allows for application of TDI [23] in a high volume arthroplasty service, can reduce the number of instrumentation trays per case from 6 to 3 and cut the related costs by 50%.

Our study is the first to report 1D digital templating by ML measurements of distal femur and proximal tibia that does not employ specific planning software but rather uses the measuring tool of the standard imaging software. Furthermore, we show that templating accuracy has good inter-rater reliability, suggesting an accurate measurement can be achieved independent of training level. This has been reported before by Hsu et al. [7], who introduced the concept of TDI for primary TKA [22].

The value of pre- and postoperative imaging in order to plan resections and control coronal alignment seems to be undisputed. However, templating the implant size of TKA is less common than in THA and the usefulness has been questioned [25]. While some reports doubted the benefit of digital templating [26, 27], multiple studies were able to show good accuracy in predicting components within one size [6, 22, 23, 28]. Traditionally, templating was performed on printed X-ray films with acetate overlays, reaching an overall accuracy within one size of 91% [4]. This has been matched or exceeded by 2D templating on digital films using specific templating software [4]. However, in order to achieve a reliable and accurate size measurement, the X-ray should be taken in a standardised fashion to obtain perfect AP and lateral images. Furthermore, a reference ball is needed in order to adjust for magnification factor. Without it, planning accuracy in TKA and THA drops markedly [6]. Our study used linear AP EOS radiography, which in contrast to conventional X-rays, exposes the patient to a lower radiation dose [15] and provides the orthopaedic surgeon with a full-body weight-bearing image without a magnification factor, even in obese patients [12]. While 2D templating requires potentially expensive software and can be time-consuming, our size measurements can be obtained using our institutions IntelePACS software and measurements take less than a minute to perform.

2D templating has been reported to predict the correct size of femoral and tibial components between 42% [29]–85% [28], and 50% [7]–90% [22], respectively. 92% [4]–100% [28–30] of femoral and 88% [22]–100% [26, 28, 29] of tibial components were templated within one size. Our 1D templating matches the reported accuracy of 2D templating with tibial component sizes off two sizes in only 3 cases (2%) and femoral in only 5 cases (3%).

Studies report intra-observer reliability in TKA templating slightly better than inter-observer agreement, ranging from good [26] to very good/excellent [7, 8]. Our study mirrors these reports. Furthermore, level of training did not influence accuracy of templating and no learning curve could be shown [7], which is in accordance to our findings.

While some studies report a high accuracy of templating on the lateral X-rays [22], our AP templating is supported by Kniesel et al. [6], who showed a higher accuracy of femoral and tibial component templating on AP than lateral views (exact size in 55% and 72% AP versus 33% and 70% lateral, respectively).

In an effort to optimise patient outcomes and reduce instrumentation trays, PSI has been developed. A reduction of trays used [9], procedure time [31], turnover time [23] and sterilisation time savings [32] have been reported. Furthermore, studies have revealed a high planning accuracy of > 90% exact sizing [8, 33–35]. Our unpublished pilot series of PSI (i.M.A.G.E®, Amplitude, Valence, France) showed equivalent accuracy levels using the implant employed for this study (Score®, Amplitude, Valence, France), where 97.5% of femoral and 90% of tibial components were exactly sized, and 100% of components were sized to within one size in 40 TKAs.

Given acceptable accuracy of templating, which was reported as > 50% [23], TDI has also been reported to reduce instrumentation trays [22], mean OR turnover time and in-room time [23]. Furthermore, TDI can reduce costs [22, 23] in TKA surgery and obviates the need for preoperative CT or MRI scans. While we did not examine the effect of TDI on OR turnover time, set-up time or in-room time, we have identified the cost-savings borne through a reduction in the number of surgical trays for TKA surgery.

### Limitations

This study must be viewed in light of its limitations. First, we included all patients with EOS scans, irrespective of deformity and rotation. This might reduce the accuracy of our templating method on one hand; however, it is a true representation of a typical caseload. Second, only one specific implant was examined, while other systems might have more available sizes and therefore reduce planning accuracy. Furthermore, numbers of instrumentation trays required for other implants may differ. Third, the cost of assembling the size and side-specific trays was not calculated, as this service was provided by the local vendor. Fourth, the cost reduction was simply based on the cost-savings per tray at our hospital and did not consider potential savings by decrease of inventory burden or reduction of labour force in sterilisation. Furthermore, we did not investigate the radiation exposure compared to standard X-rays, hence we cannot make a statement about the advantage of EOS for the patient in this regard. Finally, due to variability in the cost of EOS scans in different countries, the true cost-saving compared to conventional X-rays cannot be stated. However, based upon an average cost of $100 per scan, the cost-saving remains significant.

### Conclusions

The new 1D templating method using EOS AP imaging predicts component sizes in TKA within one size 97% of the time, allows for implementation of TDI which can halve the number of instrumentation trays and sterilisation costs. These findings might be of importance in the present environment of raising financial pressure in the healthcare industry.

## Data Availability

The datasets used and/or analysed during the current study are available from the corresponding author on reasonable request.
